# Deep Neural Networks Applied to Stock Market Sentiment Analysis

**DOI:** 10.3390/s22124409

**Published:** 2022-06-10

**Authors:** Filipe Correia, Ana Maria Madureira, Jorge Bernardino

**Affiliations:** 1 Institute of Engineering of Porto (ISEP/P.PORTO), Polytechnic of Porto, Rua Dr. António Bernardino de Almeida nº 431, 4200-072 Porto, Portugal; 1150524@isep.ipp.pt; 2 Interdisciplinary Studies Research Center (ISRC), ISEP/P.PORTO, 4249-015 Porto, Portugal; 3 Institute of Engineering of Coimbra (ISEC), Polytechnic of Coimbra, Rua Pedro Nunes, 3030-199 Coimbra, Portugal; jorge@isec.pt; 4 Centre of Informatics and Systems of University of Coimbra-CISUC, Polo II, Pinhal de Marrocos, 3030-290 Coimbra, Portugal

**Keywords:** Deep Learning, Big Data, stock data, financial markets, social networks, Sentiment Analysis

## Abstract

The volume of data is growing exponentially and becoming more valuable to organizations that collect it, from e-commerce data, shipping, audio and video logs, text messages, internet search queries, stock market activity, financial transactions, the Internet of Things, and various other sources. The major challenges are related with the way to extract insights from such a rich data environment and whether Deep Learning can be successful with Big Data. To get some insight on these topics, social network data are employed as a case study on how sentiments can affect decisions in stock market environments. In this paper, we propose a generalized Deep Learning-based classification framework for Stock Market Sentiment Analysis. This work comprises the study, the development, and implementation of an automatic classification system based on Deep Learning and the validation of its adequacy and efficiency in any scenario, particularly Stock Market Sentiment Analysis. Distinct datasets and several Deep Learning approaches with different layers and embedded techniques are used, and their performances are evaluated. These developments show how Deep Learning reacts to distinct contexts. The results also give context on how different techniques with different parameter combinations react to certain types of data. Convolution obtained the best results when dealing with complex data inputs, and long short-term layers kept a memory of data, allowing inputs which are not as common to still be considered for decisions. The models that resulted from Stock Market Sentiment Analysis datasets were applied with some success to real-life problems. The best models reached accuracies of 73% in training and 69% in certain test datasets. In a simulation, a model was able to provide a Return on Investment of 4.4%. The results contribute to understanding how to process Big Data efficiently using Deep Learning and specialized hardware techniques.

## 1. Introduction

There is currently a growing effort to find ways to take advantage of the capabilities provided by Big Data using Machine Learning (ML) for insight discovery and to improve decision making. Big Data can be defined as “significantly large datasets beyond the ability of traditional database software to store, capture, manage or analyze” [1]. The potential of these data relies on the ability to extract value from such massive data through data analytics. Deep Learning (DL) is a subfield of ML that enables performance improvements through data insights [2,3]. ML algorithms have rarely been challenged as much as by Big Data in obtaining knowledge. 

Big Data offer huge amounts of data and information that ML algorithms can work with to extract patterns or build analytical models. DL is mentioned as one of the ways to overcome some of the challenges of Big Data—in particular, feature engineering, non-linearity, data heterogeneity, uncertain, dirty, and noisy data [4,5].

Recent advances in the field of neural networks have led to the development of new, deeply structured architectures and corresponding algorithms that make them attractive for complex classification problems. In this paper, we propose that Deep Neural Networks (DNN) could play an important role in extracting value from Big Data. The goal is to study, develop, and implement an automatic classification system based on DL that can validate its adequacy and efficiency in any scenario. The use case for which this system is being developed is the classification of social network users’ sentiments towards stock market values.

A preliminary version of this paper has been published in [6]. In this version, we improve the following aspects:

- Detailed explanation of the architectures which were used to build all the Deep Neural Networks.
- In depth information about the implemented data collection and storage architectures.
- Increased coverage about the experimentation method followed.
- More additional information on the results of the experiments.

The main contributions of this paper are as follows: categorizing how to extract, transfer, load, store and pre-process Big Data; and becoming familiar with the procedure of Big Data efficiently using Deep Learning and specialized hardware techniques.

Therefore, the main contributions of this paper in the context of the current state of the art are as follows:

- Usage of Deep Learning for feature extraction from unstructured data.
- Combining different methods of Deep Neural Networks for Natural Language Processing and Sentiment Analysis to infer variations on Stock Data Value.
- Understanding how to process Big Data efficiently using Deep Learning and specialized hardware techniques.
- Stock market prediction using Deep Learning.
- Big Data extraction, management, and analysis strategies.
- Understanding how different techniques of Deep Neural Networks act on text data.
- Showing how numerical data can enrich the techniques used for sentiment prediction.
- Understanding how to extract, transfer, load, store, and pre-process Big Data.

The rest of this paper is organized as follows. Section 2 presents some background on Big Data and Deep Learning topics. Section 3 describes the materials and the methods that were applied in the development of the experiments. Section 4 describes the experimentation and evaluation methodologies used. Section 5 presents the results of the performance assessment of each implemented network, and Section 6 presents the discussion of these results. Finally, Section 7 systematizes our main conclusions and identifies some future work opportunities.

## 2. Big Data and Deep Learning Background 

In this section, we introduce the concepts of Big Data and Deep Learning. Both these concepts are relatively recent. In just the past 5 years, Deep Learning has been adopted in diverse areas, driving rapid progress in such different fields as computer vision, natural language processing, automatic speech recognition, reinforcement learning, and biomedical informatics. Big Data is a concept that has found wide adoption in recent years, as a result of the massive quantity of data generated every day. In the next subsections, we explain both these concepts in more detail. 

### 2.1. Big Data and Sentiment Analysis

Big Data is defined by various characteristics (3, 5 or 7 V’s) by different authors or organizations, each being defined to deal with Big Data contexts in a better way.

Sentiment Analysis has emerged as an important research area due to the wide use of social media platforms. As a result, a large body of literature can be found on Sentiment and Emotion Analysis. For example, in [7], a Machine Learning approach for the automatic detection of emotions from the text posted on social networks was proposed. Characteristics such as Volume, Velocity, Variety, Veracity, Variability, Value, and Visualization have an extra influence on SA models [8].

- **Volume**: The capacity to process massive data remains a critical challenge. Massive data volumes can complicate the efficiency of SA methods. Most SA methods are implemented for serial computation [9,10], which degrades performance when data reach certain sizes. Parallel processing is a possible solution that can allow adaptability to scale to dataset size. MapReduce and other MapReduce-like models are such techniques [11]. Spark is one of these MapReduce-like models capable of parallel processing [12] and is mainly aimed at Machine Learning and interactive queries. It was developed to take advantage of Resilient Distributed Datasets (RDD) to achieve performance improvements over the classical MapReduce [13]. Storage techniques such as Distributed File Systems (DFS) have been developed to reliably store very large data sets. By distributing storage and computation across many servers, the resources can grow with demand while remaining economical [14,15,16]. Data noise and inconsistency is another issue that grows with big datasets.
- **Velocity**: This refers to the speed of data generation. Big Data does not rely only on static datasets but also on data streaming in real-time (or near real-time). It is important for SA to consider not only batch processing but continuous processing to cover all possible data sources. New technologies have emerged for the various stages of Big Data, ranging from data extraction to data analysis. Tools such as Spark allow continuous data streaming [17].
- **Variety**: Big Data can have a variety of content, which can be structured or not. It is important to train SA models with different types of data to expand their ability. Another variety aspect is that text data (i.e., social network data) may not respect syntax and orthography rules and may include made-up words, strange expressions, or even emojis. These characteristics can impair systems that rely on grammars of unified languages [8].
- **Veracity**: Data must be accurate and valid; otherwise, using corrupted data will result in an invalid model that cannot provide reliable insights. Different sources of data can cause some issues in terms of data quality and reliability. Data from social networks contain considerable noise, which can result in meaningless results [8].
- **Value**: Value in data can be hidden and difficult to find. Sentiment Analysis that does not adapt to the nature of data being processed may find it difficult to reach the right level of performance. The real value lies in selecting the best model to follow for analysis and the best data as well [8].
- **Variability**: Continuously produced data may continuously change meaning. For example, in social network data, a user’s opinion may change with time, and users may continuously update their intents. Being able to adapt to these changes is very important. Another issue is that words may have different meanings depending on their context. To address this, contextual dictionaries may provide some support [8].
- **Visualization**: The value and output must be seen and understood. Data visualization allows complex analysis to be displayed in simple ways. SA models are still lacking in terms of visualization. There are other interesting aspects than the number of messages being processed that should be taken into consideration [8].

Sentiment Analysis is usually performed based on textual data. This kind of data cannot be used in its raw form. In [18], a five-step plan is proposed from data extraction to classification:

1. Preprocessing, by tokenizing text, removing noise and unnecessary tokens, and then finding the root of each token.
2. TF-IDF Feature Extraction, where sentences are transformed into a Bag of Words by counting and normalizing the tokens and then weighing the tokenized words using the term frequency-inverse document frequency. TF is the term frequency of a token and IDF is the number of phrases containing a certain term.
3. Word Embedding Feature Extraction—a technique of representing a word into a fixed-size vector with the help of contextual information. *GloVe* [19] and *Word2Vec* [20] are widely used frameworks for this effect.
4. Feature Fusion, combining the results of TF-IDF that have syntax and context information with the results of Word Embedding Feature Extraction.
5. Classification, using machine learning classifiers such as Support Vector Machines (SVM), Random Forrest (RF), K-Nearest Neighbors (KNN), or even Deep Learning (DL).

More proposed Sentiment Analysis methods include the use of complex input structures, such as in the Improved Word Vector [21]. This structure combines word embedding vectors based on NLP techniques, lexicon-based approaches (lists of phrases and words that have polarity scores), word position algorithms as well as *GloVe* and *Word2Vec* methods. The result is a complex structure that can then be provided for a Deep Neural Network. 

### 2.2. Deep Learning

Deep Neural Networks (DNN) are more complex versions of Artificial Neural Networks (ANN). ANN is an umbrella term, the name of which was inspired by how biological neural processing works, simulating the way the brain processes information [11]. DNNs consist of multilayer interconnected nodes containing more than one layer of hidden layer nodes. Multiple hidden layers enable parameter learning and classification in the same network. DNNs get their name from the number of hidden layers they have. DNNs can be trained in a supervised training mode, where the target attribute to be predicted or classified is present in the training data, or in an unsupervised mode, where the training data are automatically generated from unlabeled data with little human intervention [4].

Deep Learning is used extensively in image and video processing for image recognition systems because of its ability to resolve these complex data types into a series of nested, simple mappings, each described by a single layer of the model [2]. Convolutional Neural Networks (*CNN*s) are mainly used for image and video processing and are a subtype of DNNs inspired by the visual cortex of animals. They break down an input into smaller parts (e.g., a 32-pixel image is analyzed 5 pixels at a time) to keep the size of the network manageable [12]. Table 1 shows some DNNs and their main applications.

DNNs such as Auto Encoder are unsupervised learning algorithms used for efficient dimensionality reduction. They are ANNs with multiple hidden layers used to learn features before proceeding to classification, all in the same network [13]. 

Recursive Neural Networks (RvNNs) are also DNNs. What makes them recursive is their architecture, which allows the recursive application of the same set of weights within a structural environment. RvNNs have a special oblique tree structure that allows RvNNs to work well in NLP [22].

*CNN*s are hierarchical architectures that are well suited for position-invariant feature extraction [23]. *CNN*s are not much associated with NLP per se, but when used with position encoders, they have been shown to be useful for NLP classification. *CNN*s use convolutional layers, pooling layers, and *Fully Connected layers* to process data [24].

Recurrent Neural Networks (RNNs) are a subtype of RvNNs with a specific structure. Since NLP depends on the order of words or sentences, it is useful to have a “memory” of previous elements when processing new elements due to backward dependencies (e.g., the semantic meaning of a word may depend on the words before or after it). RNNs achieve this by combining the outputs of two layers to allow the analysis of phrases in both forward and backward directions, also known as Bidirectional Recurrent NN [25]. Another mechanism by which the capabilities of RNNs can be enhanced for NLP is the use of Long Short-Term Memory (*LSTM*), in which single recurrent nodes are exchanged for multiple individual neurons (interconnected nodes) that are linked in such a way that they retain, forget, or expose important information [22]. The Gated Recurrent Unit (*GRU*) is a subcategory of *LSTM* that does not contain a separate memory cell [26]. An *LSTM* cell controls the exposure of its memory to the other cells in the network, while the *GRU* always exposes all memory.

Another characteristic that can allegedly improve the performance of Deep Learning on text data is the use of an attention mechanism. These mechanisms allow an NN to focus on certain aspects of the input and filter out noise [27]. 

These different NN techniques have been used in various works with some success. Using *CNN*s, *LSTM*, and *GRU* alone gave good results with social network data [28]. From individual implementations, hybrid models were developed that combined *CNN*s with both *GRU*s and *LSTM*s. For this particular use case, the application of a *CNN* for sentiment analysis and an *LSTM* for numerical data analysis proved to be successful [29]. This is the basis on which the work in this paper has been developed.

## 3. Materials and Methods

The proposed approach consists of the following three phases:

- Phase 1: Select an existing dataset that had already been processed and used in other works. These data are used to train seven networks, each with different combinations of neural layers (*CNN*, *LSTM*, *GRU*, *CNN*-*LSTM*, *CNN-GRU*, *CNN-BiGRU* and *CNN-LSTM* with Stock Indicators). These networks are then used for sentiment analysis related to stock value changes.
- Phase 2: Collect data from different social networks and store them in DFS. These data are then used to test the models obtained in the training phase.
- Phase 3: Two trading simulations based on the two best models in the test phase. To validate if there is any value in the information which is used for testing and training, there is a baseline added to the simulation where 1000 USD is invested on the first day of the simulation and only removed on the last day, following the movement of the markets.

The next sections describe the experimental setup, Deep Learning architectures, data collection, storage, and finally, the methodology used in the experiments.

### 3.1. Experimental Setup

For the development of the proposed algorithms, we used Python version 3.8 running on a Windows AMD Ryzen 5 3600 computer with 16 GB of memory, an NVIDIA RTX 3070 GPU, and 4 TB of disk space. The implementation of the different algorithms was based on TensorFlow in Keras and assisted by Jupyter Notebooks. 

For the proposed Big Data storage architecture, we used the Windows Subsystem for Linux (WSL), where Spark 2.3 and Hadoop 3.3.0 were installed and configured to work with each other. 

### 3.2. Deep Learning Architectures

Keras was selected for the implementation of the Deep Learning algorithms. As this is a Python-based framework, Jupyter Netbooks were used as a workbench for the development and testing of the different architectures, as they allow small snippets of the code to be executed, permitting the debugging of the implemented code.

#### 3.2.1. *CNN*-Based Architecture

**Figure 1:**
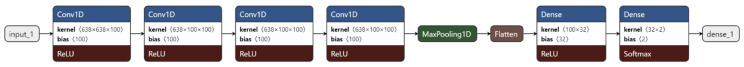
Implemented *CNN*.

The implemented *CNN* architecture shown in Figure 1 was based on the Improved Word Vector Architecture [21]. The name *Improved Word Vector* comes from the fact that the input to a DNN is a tensor made up of various word embedding techniques, such as Natural Language Processing, Lexicon-Based approaches, word position, *Word2Vec*, and *GloVe* methods. The Lexicon2Vec array, which is a Natural Language Processing technique that outputs the polarity of words, was implemented using six different lexicons as employed in the original work [21]. The Part-Of-Speech Vector tagging, which consists of the context of each word in a phrase as a function of its neighbors, was implemented using a Python framework and concatenated to the *Word2Vec/GloVe* array. The word position comprises simply a vector that contains the location of a word relative to the start and the end of a phrase. The *Word2Vec/GloVe* embedding was done using pre-existing bags of words mapped to arrays with size 300. This was done by mapping words to the correspondent array in *Word2Vec*, and then, if not present in *Word2Vec*, in *GloVe*. If neither of these dictionaries contained the word, a random array of size 300 was then generated.

The input for this model was a multi-dimensioned tensor that concatenates the embedding of each word, using the methods referenced above. To make it possible for the Neural Network to process these inputs, each sentenced then had to be padded. This resulted in a tensor with a standard size for each dimension.

The Neural Network itself consisted of an input layer, followed by four convolutional layers (one more than the one employed in the original work) all with the *Rectified Linear Unit* (ReLU) activation. The next layer performed *Max Pooling,* which pools the highest values from the convolutional layer output. A flatten layer reduced the dimensionality of the tensors in the network. Following this were two *Fully Connected layers*, one with 100 units and a ReLU activation that was then fed to a simple *Dense layer* with only 2 units and a *Softmax activation*. The output was a two-dimensional array with the probability of a phrase being 0 or negative sentiment and 1 corresponding to positive sentiment. Figure 1 contains a graph describing the implementation flow. 

For the hyperparameter definition, some tunning effort was involved to find the best parameters, which consisted in testing different configurations and selecting the configuration with the best accuracy and loss results. Table 2 has the list of the hyperparameters used in the definition of this network. In total, 200 epochs took place inside two cycles as per the *nCV* training methodology. The outer cycle was repeated 5 times—the same as the inner cycle.

#### 3.2.2. *LSTM*-Based Architecture

**Figure 2:**
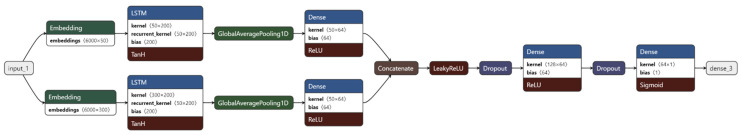
Implemented *LSTM*-NN.

The implemented *LSTM* Neural Network (in Figure 2) consisted of two distinct inputs. The first was based on the *GloVe* embedding method which, as in the *CNN* architecture, consisted of arrays of 300 elements, as opposed to the architecture used in the original work that contained only 100 elements. The second input was based on semantic word embedding made up of arrays of 50 elements. 

The constructed Neural Network was built with embedding layers that worked as lookup tables, where each entry mapped to an embedding array. To do this, it was necessary to build a vocabulary, which was a map of numbers, each attributed to a different word. Only the 10,000 most common words were included in the vocabulary. In the *GloVe* embedding layer, the embedding arrays for each word of the vocabulary were entered into a list and ordered according to the order of the vocabulary. This list was then assigned as the weights of the embedding layer, which could not be trained in order to not change the pre-assigned values. The embedding layer, however, was trained, as it was built as an empty layer. 

Both branches of embedding were assigned with an *LSTM* layer that connected to a fully connected *Dense* layer. There was a need for a *Global Average Pooling* between the dense and *LSTM* layers due to the dimensionality of the data. The *Dense* layer outputs were then concatenated and passed to two more fully connected *Dense* layers, one of them a Leaky ReLU layer. The output layer was also a *Dense* layer that differed from the one implemented in [30], as it was a binary sigmoid activated layer, producing only the values 0 and 1 for positive and negative sentiments, respectively. There were also two dropout layers that were added to avoid the overfitting of the model. Figure 2 contains a graph describing the implemented *LSTM* Neural Network.

The hyperparameters were not listed in the original paper, so there was some tunning involved to find the best parameters, which consisted in testing different configurations and selecting the configuration with the best accuracy and loss results. Table 3 has the list of the hyperparameters used in the definition of this network. The 150 epochs took place inside two cycles as per the *nCV* training methodology. The outer cycle was repeated 8 times and the inner cycle 10.

#### 3.2.3. *GRU*-Based Architecture

**Figure 3:**
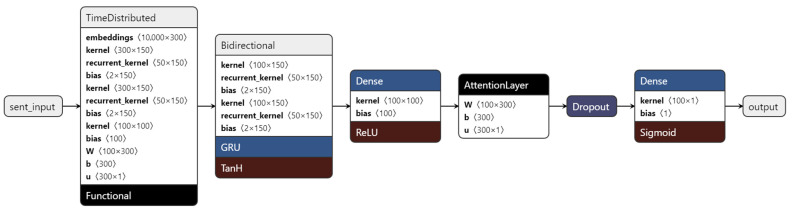
Implemented *GRU*-NN.

The *GRU* implementation shown in Figure 3 used an Attention Layer. The input, much like the NN in the previous section, was obtained by embedding the words using an embedding layer with embedding arrays from *GloVe*. However, this needed higher dimensionality, as there were two distinct encoding moments: one for words and the other for sentences. Consequently, we added a size 1 dimension level to the tensor to account for this. This architecture was developed for whole bodies of text as opposed to the single sentences obtained for the training phase dataset.

The Time Distributed layer was added to make it possible to analyze lower dimensions of the input—iIn this case, the word dimensions, and inside it the embedding, the bidirectional word *GRU,* and attention layers, including also a *Dense* fully connected layer with ReLU activation. The output of the Time Distributed layer was then passed to a similar network as the one it contained for sentence level encoding. There was also the addition of a dropout layer to avoid overfitting. The last *Dense* layer consisted of a sigmoid, which resulted in a binary output, where 0 is negative sentiment and 1 is positive sentiment. Figure 3 contains a graph illustrating the implementation.

The hyperparameters were not listed in the source paper and were obtained by tunning the model, which consisted in testing different configurations and selecting the configuration with the best accuracy and loss results. Table 4 has the list of the hyperparameters used in the definition of this network. The 150 epochs took place inside two cycles as per the *nCV* training methodology. The outer cycle was executed 10 times—the same as the inner cycle.

#### 3.2.4. Convolutional and *GRU*-Based Architecture

**Figure 4:**
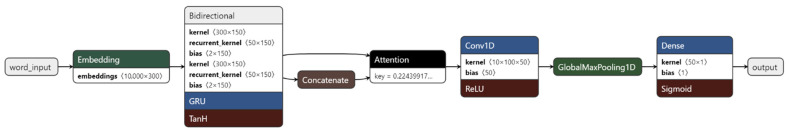
Implemented *GRU*-*CNN* with bidirectional *GRU*.

**Figure 5:**
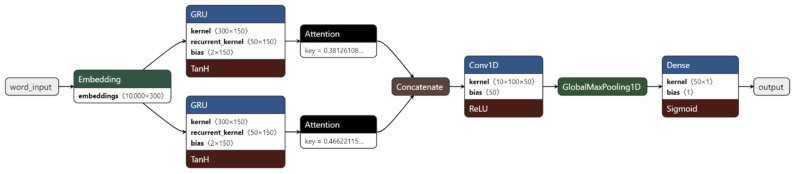
Implemented *GRU*-*CNN* with separated forward and backward encoding.

Two Neural Networks were built using a combination of Convolutional and *GRU* nodes:

- One with a bidirectional *GRU* (Figure 4) (*BiGRU*).
- The other with separate forward and backward-oriented *GRU*s (Figure 5), which changed the attention layer inputs and the number of attention layers needed.

There was a need for two networks in the separate *GRU*, where each gave attention to either the forward *GRU* or the backward *GRU*, and one only for the Bidirectional *GRU,* which resulted in the attention mechanism being applied only to the bulk of the *BiGRU* outputs. 

The embeddings were obtained by using *GloVe* encoding [31]. The convolutional layers were implemented with the ReLU activation function in both networks, and the *GRU*s were implemented with TanH activation [32].

The output layer was a *Dense* Fully Connected layer with a sigmoid output that output 1 if the sentiment was positive and 0 if it was negative. Figure 4 and Figure 5 contain graphs representing the two implementations.

The hyperparameters were not included in the source materials, so they were selected by testing different configurations and selecting the best performers. Table 5 shows the list of the hyperparameters used in the definition of both networks. The 150 epochs took place inside two cycles as per the *nCV* training methodology. The outer cycle was executed 10 times—the same as the inner cycle.

#### 3.2.5. Convolutional and *LSTM*-Based Architecture

For the *CNN* and *LSTM* Neural Networks, two different concepts were tested:

- The first one, much like the previous implementations, consisted of using only the news data.
- The second approach used numerical information in the form of stock values to enrich the results obtained from the analyzing the same news data [29].

**Figure 6:**
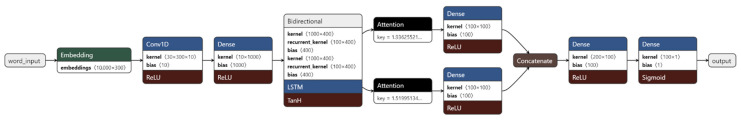
Implemented *LSTM*-*CNN*.

Figure 6 presents the first approach, which used an embedding method such as *GloVe* and passed the outputs to a convolutional layer. Next, data were passed through a fully connected *Dense* layer and in the end to a bidirectional *LSTM* (*BiLSTM*) which was connected to two different attention mechanisms for forward and backward *LSTM* processing. The output was a sigmoid *Dense* fully connected layer, producing a binary result: 1 for positive sentiment and 0 for negative. Figure 6 contains a graph illustrating the implementation.

The source code included the hyperparameter information, but further testing with different configurations resulted in the parameters in Table 6. The 150 epochs took place inside two cycles as per the *nCV* training methodology. The outer cycle was executed 8 times and the inner cycle 10. 

**Figure 7:**
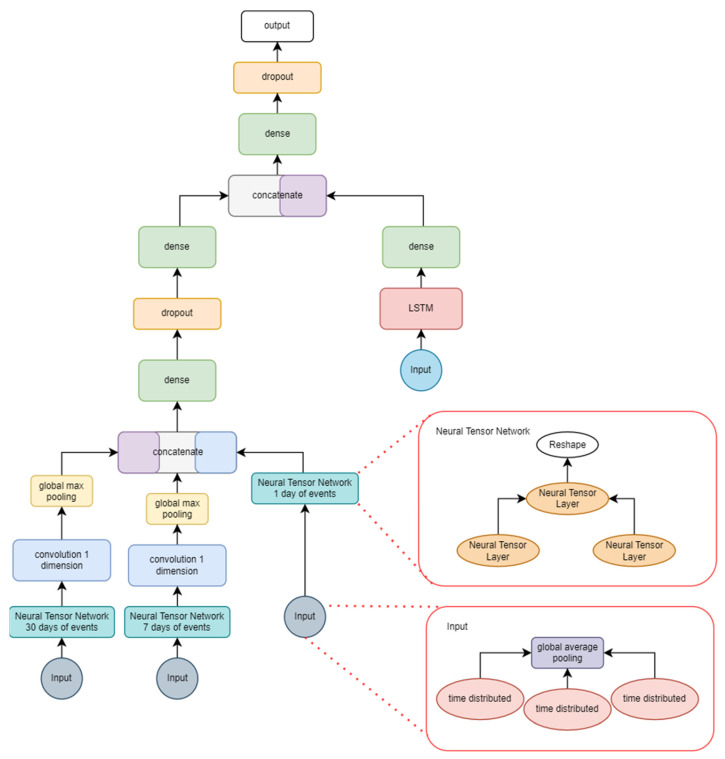
Implemented *LSTM*-*CNN* with stock data.

Figure 7 contains the graph of the second approach, which used the same dataset that was used in this paper for training all the implementations. Data consisted of date-marked headlines from 2008 to 2016 extracted from Reddit. However, this approach changed the input format needed, as it contained three different inputs—one for each timeframe (month, week, day) of headline data—and there was an added input of the DJIA stock values (open, close, high, and low) as well as seven different indicators based on these values which are used by stock traders in their investment strategies.

Table 7 contains the hyperparameters used in the training of this model. The 150 epochs were repeated 1990 times, since the model used an incremental training model, where each cycle corresponded to a day in terms of training data. The batch size was 1; however, that input contained three distinct structures—one of which had data for the last 30 days, another for 7 days and the last for the present day—due to the way in which data were structured for the input of this network. 

The implementation in Figure 7 shows the Neural Tensor Layer implementation—one for each time level (month, week, and day). After a global pooling layer, the NN fed convolutional layers which were then concatenated. Another concatenation occurred with the result of the *LSTM* layer, which processed stock indicators. Those indicators contained data for the last 30 days. The output of this neural network was binary, where 1 meant there was a positive sentiment and 0 a negative one. The hyperparameters were defined according to the parameters proposed in the source work [29], as there was no significant variation in results with the different configurations tested. Due to the nature of this architecture being based on daily, weekly, and monthly data, a time-based approach was taken for training instead of randomly shuffling the data.

### 3.3. Data Collection and Storage

The Deep Learning models were trained using a curated dataset, containing Reddit news headlines with the sentiment value, which was inferred from the difference between closed and open values of the stock markets for the DJIA index [29]. This dataset was already used in other research papers, so there was no need to collect and prepare any data for the training phase, as they were in a text format, ready for use. The test phase, however, relied on data collected from social networks and stock data trackers. 

For the validation phase, in which there was an in-depth performance comparison of the different models, external sources were used. The objective was to understand how each model reacts to different scenarios. 

**Figure 8:**
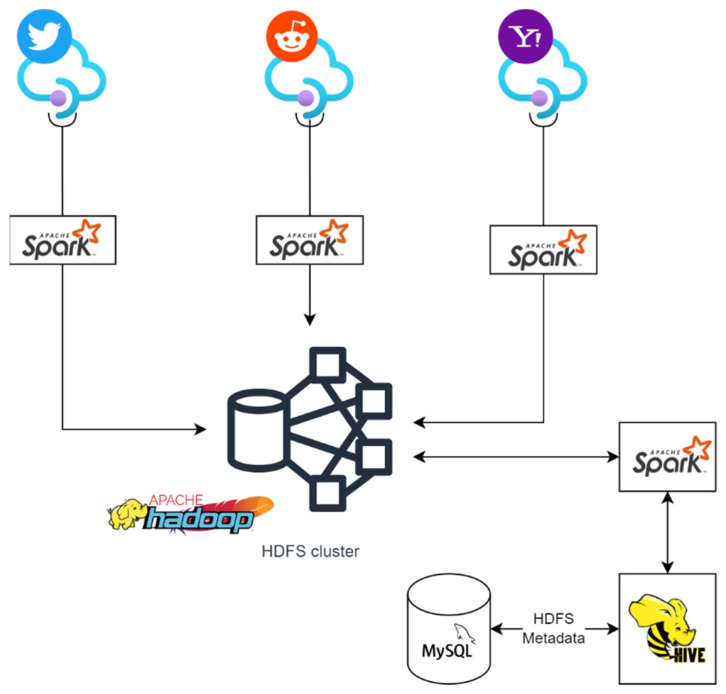
Big Data storage architecture.

Using Big Data technologies, such as Spark, Hadoop/HDFS, and Hive, it was possible to implement a data warehouse. Data were extracted from Twitter and Reddit on an hourly basis, and the Yahoo! Stocks API produced the daily stock values for four different indexes (DJIA, BTC, AAPL, and TSLA). In Figure 8, we can see the resulting architecture for the Big Data storage. Three different applications queried three different APIs (Twitter, Reddit, and Yahoo! Finance) and wrote the resulting responses to the HDFS cluster using Spark as the engine for the write operation. Hive stored the metadata of the tables in HDFS in a MySQL database, which allowed it to keep track of tables, columns, etc. Spark acted as the engine for Hive as well. Hive queries were executed within Spark and their result then sent back to Hive. These queries helped to update the metadata tables in MySQL.

**Figure 9:**
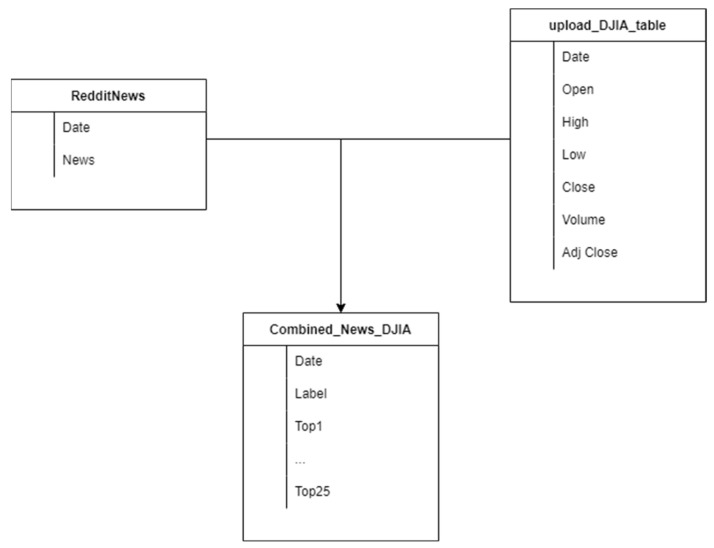
Training dataset.

In the training phase of the binary classification problem, we used the dataset described in Figure 9. This dataset contained three different tables [29]:

- One including Dow Jones Industrial Average (DJIA) information, with the Date, Open, High, Low, Close, Volume and Adjusted Close attributes for the DJIA index. This table had 1990 lines.
- Another comprising Reddit news headlines, containing only Date and News columns, with 74,377 lines.
- A table that combined the information of the previous two, containing 1990 rows, each comprised of the date, the sentiment (1 for positive, 0 for negative) of the day, and up to 25 different news headlines, extracted from Reddit for each date.

These data were transformed into a structure that mapped each new headline to a single row; thus, there were various rows for a specific day, each with a particular news headline.

**Figure 10:**
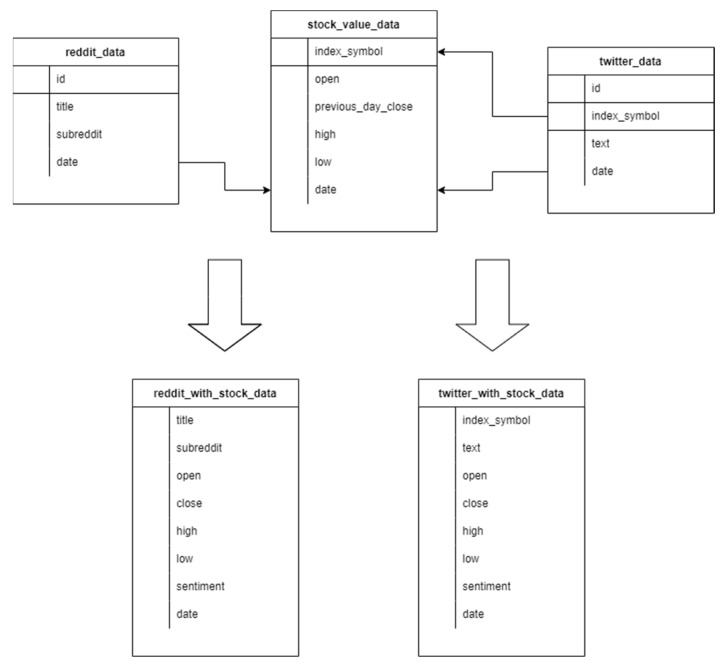
Test dataset.

For phase 2 of the binary classification problem, the subsets in Table 8 were used, which were produced from the datasets in Figure 10. From three different sources, stocks, tweets, and Reddit titles were collected. Stocks were combined with both Reddit and Twitter data. From Twitter, we extracted tweets mentioning four different Stock Indexes (AAPL, BTC, DJIA, and TSLA). From Reddit, we extracted post titles from three different Subreddits that discuss stock values.

Each tweet was related to the stock index it represents, resulting in four different tables. Reddit data were all related to the DJIA index, so each subreddit was used to differentiate data into datasets. The profit/loss for each day was calculated by subtracting the closing and the open prices for the stock data. When the result was a negative value, the sentiment for all sentences of a certain day was labeled as negative. When the result was positive, the sentiment was labeled as positive.

All networks, apart from *CNN*–*LSTM* with Stock Indicators (*CNN*–*LSTM* SI), were trained and tested using sentence data as input, since this was the required input. The difference in the training method for *CNN*–*LSTM* SI was due to the usage of numerical indicators, which contained the open, closed, high, and low values of stock indexes. Appended to them were a list of analytic formulas used as a guide by professional traders, which are presented in Table 9.

Different data sources show how algorithms behave under different conditions. This is related to the variety of characteristics of Big Data in the way that as data may vary in various forms and come from different sources, they can also have different contexts. Having multiple sources and multiple algorithms to test can give a clearer representation of how and if Deep Learning can overcome this specific aspect.

### 3.4. Development Methodology

**Figure 11:**
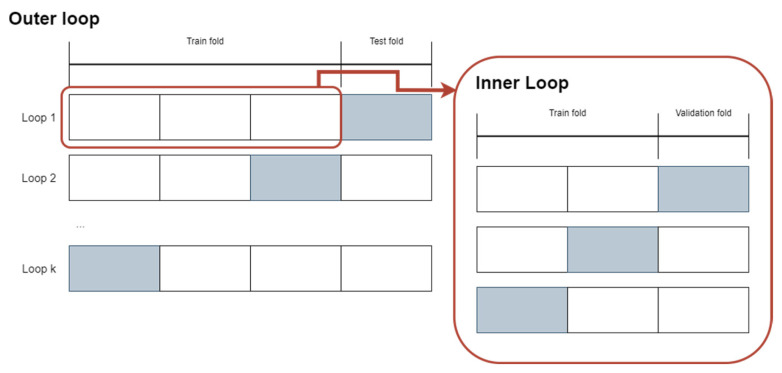
Nested Cross Validation methodology.

Nested Cross Validation (*nCV*) is the technique that was used to create and train the eight networks. This approach iterates over data several times, consisting of two nested cross-validation cycles [33,34], where the inner cycle outputs the model’s best hyperparameters, as shown in Figure 11. Data must be dynamically divided into training and validation sets to be fed to the Tensorflow framework, which uses the data on its feedback and feedforward loops, delivering a better fit during training. At the end of the inner loop, the selected model is used to classify the test data, which allows us to calculate the accuracy and loss of the model.

**Figure 12:**
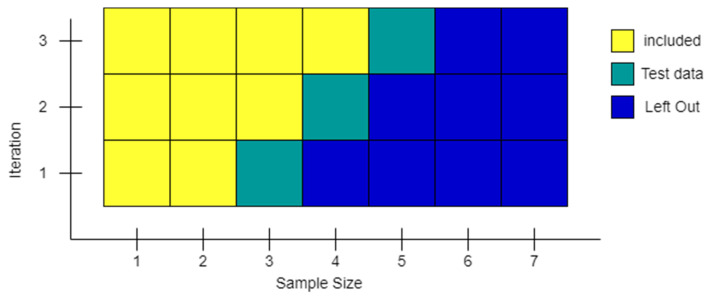
Nested Cross Validation for a time series (adapted from [35]).

Due to its specific nature, the *CNN*–*LSTM* SI network was trained with an adapted *nCV* method. The input of this algorithm consists of four different multi-level matrices. The first contains the record data for the day to be analyzed. The second and third contain aggregations of the data for the previous week and month of record data, respectively. The last one contains the stock value indicators. This means that the algorithm must be handled like a time series classifier, as shown in Figure 12. Using this behavior, training is performed incrementally, with each iteration containing all the data from the previous iteration (if it is possible to keep it in memory). Apart from the *CNN* algorithm, all the others have been implemented with embedding. This allows the creation of a vocabulary of the *n* most popular words in a dataset, with each word assigned to a number. *GloVe* can then be used to convert these numbers into arrays. The arrays consist of relationships between words defined by numbers. This conversion takes place at runtime so that the huge data arrays do not have to be kept in memory.

*CNN* uses a complex input structure, comprised of various embedding techniques, such as *GloVe*. The embeddings translate to numerical data the metadata associated with the position of words in a sentence, meaning, and other factors [28]. This method provides context to the neural networks with the disadvantage of a higher memory requirement.

To select the best hyperparameters for each implemented algorithm, each of them was run several times with different configurations. The configuration with the best results was used in terms of hyperparameters.

The use of these distinct implementations allows a broader view of how different Deep Learning techniques affect the results of Big Data analysis. It is possible to see that some techniques have advantages and disadvantages, both in terms of training time, accuracy, and other metrics.

## 4. Experimental Evaluation 

This section describes the experimentation and evaluation methodologies used in the experiments.

### 4.1. Experimentation Methodology

**Figure 13:**
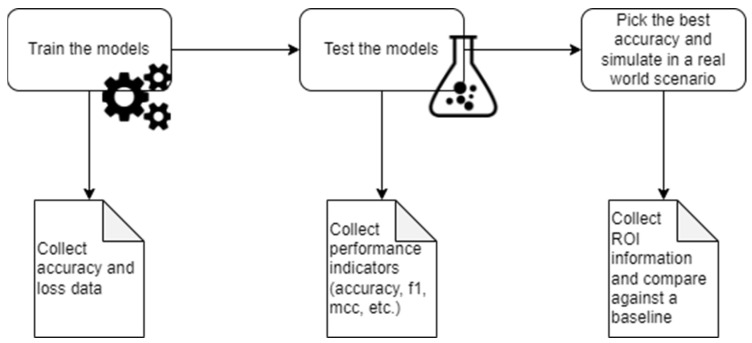
Experimentation methodology.

The experiments were divided into three distinct phases, as shown in Figure 13:

- The training phase output the accuracy, loss, and training time of each algorithm. In this phase, only the Reddit Stock Headline dataset was used, as it had already been pre-processed.
- The second phase consisted of the evaluation of the models with different test data. These evaluations output the accuracy, precision, sensitivity, fallout, specificity, F1 score, and MCC. The datasets used in this phase were the AAPL, BTC, DJIA, and TSLA created from Twitter data, as well as Stocks, Stock Market, and Wallstreetbets from Reddit, plus an extra one with a mix of all Reddit data.
- The third and last phase consisted of the real-world scenario simulation of the best model on two distinct datasets.

All the models except the *CNN*–*LSTM* with stock indicators evaluated the sentiment of phrases in relation to the stock market individually. *CNN*–*LSTM* with stock indicators evaluates the aggregate sentences for a whole day, as well as the sentences before that day, so it is the only model ready for real-world trading simulation. For the other models, in case they ended up being selected, the simulation was done by averaging the predicted sentiment of all sentences. Since sentiment is a seen as a binary value, if the average is higher than 0.5, then it is considered as positive sentiment; otherwise, it is considered negative. 

### 4.2. Evaluation Methodology

Machine Learning has a set of key performance metrics that can be used to self-assess a model. These can be extracted from a confusion matrix. A confusion matrix is a way of capturing and extracting the significance of predictions and true values. False positives (FP) are values that were predicted to be positive but are negative. False negatives (FN) are values that were predicted to be negative but are positive. True positives (TP) are values predicted to be positive and are positive. True negatives (TN) are predicted to be and are negative. 

Several indicators can be derived from these metrics, such as **Accuracy** (**ACC**), the ratio of correct predictions to total predictions (1):

(1) $$\mathrm{ACC}\;=\frac{\mathrm{TP}\;+\;\mathrm{TN}}{\mathrm{TP}\;+\;\mathrm{TN}\;+\;\mathrm{FP}\;+\;\mathrm{FN}}$$

**Precision (P)**, the positive predictive value, proportion of positive identifications which was correct (2):

(2) $$P\;=\frac{\mathrm{TP}}{\mathrm{TP}+\mathrm{FP}}$$

**Recall or Sensitivity (S)**, the true-positive rate, the proportion of actual positives which was correctly identified (3):

(3) $$S\;=\frac{\mathrm{TP}}{\mathrm{TP}\;+\;\mathrm{FN}}$$

**Fallout (F)**, the false-positive rate, the ratio between the number of negative events wrongly categorized as positive (false positives) and the total number of actual negative events (4):

(4) $$F=\frac{\mathrm{FP}}{\mathrm{FP}+\mathrm{TN}}$$

**Specificity (SP)**, the true-negative rate, the proportion of negatives that are correctly identified as such (5):

(5) $$\mathrm{SP}\;=\frac{\mathrm{TN}}{\mathrm{TN}\;+\;\mathrm{FP}}$$

**F1 Score** the harmonic mean of precision and sensitivity, which is defined as (6):

(6) $$F1\;\mathrm{Score}\;=\frac{2\mathrm{TP}\;}{2\mathrm{TP}\;+\;\mathrm{FP}\;+\;\mathrm{FN}}$$

**Matthews Correlation Coefficient** (**MCC**), a performance metric which focuses on all four quadrants of a confusion matrix. MCC can be advantageous as it only rewards models when they have good performance in all quadrants. It produces values between −1 and 1, where 1 shows complete agreement of correlation and −1 complete disagreement. If the result is 0, the prediction is said to be uncorrelated with the ground truth (7).

(7) $$\mathrm{MCC}\left(\theta \right)=\frac{\mathrm{TP}\;\times \;\mathrm{TN}\;-\;\mathrm{FP}\;\times \;\mathrm{FN}}{\sqrt{\left(\mathrm{TP}+\mathrm{FP}\right)\left(\mathrm{FP}+\mathrm{FN}\right)\left(\mathrm{TN}+\mathrm{FP}\right)\left(\mathrm{TN}+\mathrm{FN}\right)}}$$

The use case of this work is related to stock market data, which is why an investment simulation was made for each trained model. Once the two models with the best performance in the test data were identified, they were used to simulate an investment strategy. The investment strategy was to start with 1000 USD invested on days when the model predicted positive sentiment. If the model predicted a negative sentiment for a given day, all the money invested was withdrawn. If a positive sentiment was predicted, all the money was invested despite there being a loss or a gain compared to the original investment value.

## 5. Experimental Results

The experimental phase contained the results of the developments and provided information on how the Deep Neural Networks interact with the data. The training phase yielded the accuracy, loss, and time of training of each DNN. The test phase yielded a more complete set of metrics by using the best models produced in the training phase and exposing them to other data sources and contexts. 

The aim of this process is to understand the performance of different techniques and architectures for sentiment analysis on textual data. The results are explained in the next sections. 

### 5.1. Training Phase

The training phase was performed using the *nCV* method described before and was conducted by applying the method to the data presented in Figure 9. The networks were trained with all data available at the beginning (divided into train and validation), except for *CNN*-*LSTM* SI, which was trained in steps as a timeframe. Each step incremented the data used in the model. When the computation limits were used due to the input structure size, then data were added, and older data began to be removed from the input. The objective was to take advantage of the “memory” provided by the *LSTM* layer. 

The accuracy and loss obtained at the end of training for all models are shown in Table 10. The values are the average of accuracy and loss obtained in the end of the training using the Nested Cross Validation method. The table also contains the training time of each network. 

Accuracy was shown to be extremely good in *CNN-LSTM SI* compared to the other models, possibly due to the network input (a mixture of NLP and numerical stock value data). *CNN-LSTM SI* achieved the highest accuracy, but at the same time required much more time for training.

*CNN-LSTM* (the model without stock index data) shows the worst results related to loss. This means that it fails to hit significantly more predictions than the other algorithms. For all other algorithms, the differences seem to be less significant. The next section focuses on the models that emerged in the training phase. The tests section is about exposing these models to new data from different sources and with different contexts.

The *CNN*-*LSTM* with stock indicators is capable of a greater accuracy but at the same time takes much longer to train. In terms of loss, *CNN*-*LSTM* (without stock index data) shows the worst results. This means it fails in considerably more predictions than the other algorithms. All the other algorithms’ metrics have low significant difference. 

### 5.2. Test Phase

In the test phase, a network of each type with the best accuracy is selected to perform predictions in different datasets in similar contexts. The test consists of registering the prediction against the true value. With this information, the confusion matrix of each analysis is then built. 

The list of indicators used in the test phase was extended in relation to the training phase. Table 11 shows the average of each extracted indicator, obtained by averaging the results of the different confusion matrices obtained for each network type. 

**Figure 14:**
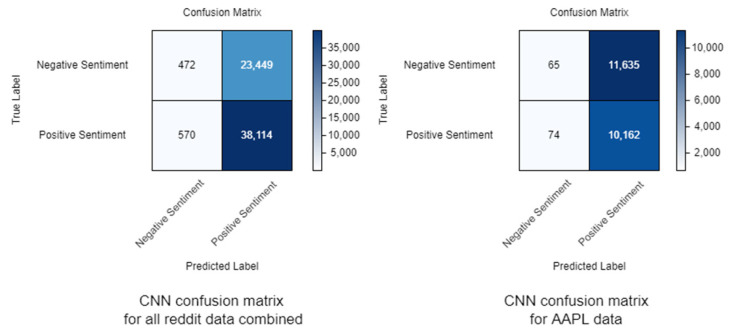
Two examples of *CNN* confusion matrices.

*CNN* shows the highest average accuracy and *CNN*-*LSTM SI* the lowest - the opposite of the results obtained in the training phase. The results obtained with the *CNN* show that it is strongly biased towards positive sentiment (Figure 14). Specificity is very low, possibly due to a low proportion of negatives being correctly identified as such. This is confirmed by the results for precision and sensitivity (the proportion of positive identifications that were correct and the proportion of actual positives which was correctly identified respectively). Fallout is also related to this, as fallout is the ratio between the number of negative events erroneously classified as positive (false positives) and the total number of actual negative events. The analysis of the results should not be limited to accuracy alone but should also consider the other metrics.

**Figure 15:**
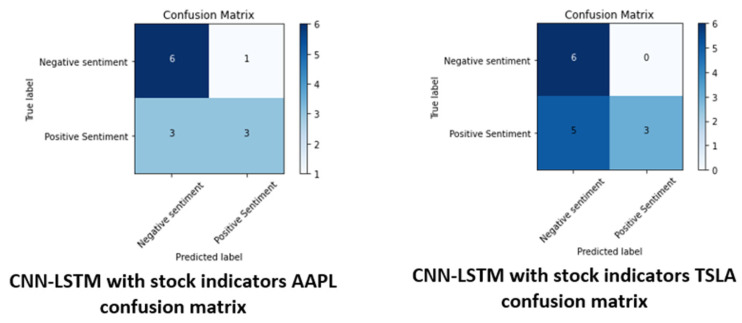
Confusion matrices with the best results.

The best accuracy results, excluding the biased *CNN*, are presented by the *CNN*-*LSTM* model. Although it has the lowest average value, *CNN*-*LSTM SI* achieved the best results in classifying the AAPL dataset, achieving 69% accuracy and 64% accuracy on the TSLA dataset, which may be due to the use of numerical data for the stock indicators. These results are shown in Figure 15. *CNN*-*LSTM SI* was also the model with the highest variance for the classification indicators. When using the MCC formula, it gets the highest correlation coefficient, while *CNN* gets the lowest—similar to the training results.

Figure 14 and Figure 15 show different values due to the way the algorithms process data. *CNN* processes each sentiment line individually, whereas *CNN*-*LSTM SI* processes an aggregate of the sentiment data for each day. 

**Figure 16:**
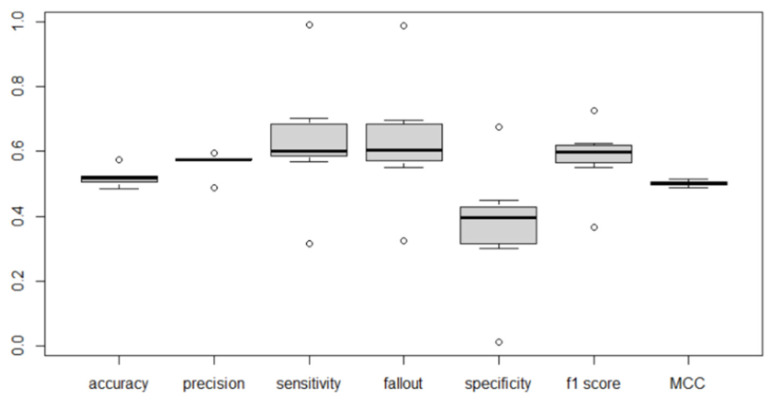
Test indicator distribution.

Table 11 is constituted by the mean values of the experiments for each metric and for each algorithm. Figure 16 shows the values in Table 11, as well as the distribution of those values. 

Specificity is very low, possibly representing a low proportion of negatives that are correctly identified as such and has also a wide variance. This may have been a result of the bias to positive predictions in some models. This is supported by the results for both precision and sensitivity, the proportion of positive identifications that were correct or the proportion of actual positives that were correctly identified. Fallout also shows a relation to this, since fallout is the ratio between the number of negative events wrongly categorized as positive (false positives) and the total number of actual negative events. Since there is a bias to positive sentiment, fallout tends to be higher, meaning there are more negatives that were classified as positive. The F1 Score is intrinsically attached to the value of precision and sensitivity, since it is the harmonic mean of the two metrics.

The algorithm selected to run the investment simulation in the following section is based on the MCC metric, as the results obtained in the accuracy metric favor a biased model, while the MCC penalizes it (this is supported by the other metrics). This indicator has a statistically significant different accuracy. *CNN*-*LSTM* with stock indicators performed the best on the MCC metric. 

The selected datasets for the simulation are those with the best performance for the selected model, which incidentally are the best results from all the samples. The first dataset is the AAPL stock data information—the dataset with the second best MCC and the best accuracy. The second dataset is the one with the best MCC result and second-best accuracy - the TSLA dataset. The next section focuses on the simulation of an investment strategy using the two mentioned dataset’s results.

### 5.3. Simulation Phase

This section covers the simulation of an investment strategy using the *CNN*-*LSTM* SI model’s prediction results on the AAPL and the TSLA datasets. This simulation was conducted by applying the best *CNN*-*LSTM* SI model obtained to different data from those used in the test phase, for AAPL and TSLA datasets. For each day, based on the data, the model outputted 1 or 0—positive sentiment or negative sentiment, respectively. With that output, the following simulation rules are defined:

- The starting balance is 1000 USD.
- If the predicted sentiment is 1, the full balance is invested.
- If the predicted sentiment is 0, the full balance is removed from the investment and placed into savings.
- Each time the money is invested, it is done so completely, leaving savings empty.
- In the end, the profit or loss is obtained by calculating the difference between the remaining balance and the initial investment.
- The Return on Investment (ROI) is the profit or loss divided by the initial investment.
- As a baseline, 1000 USD is invested in the first day and never removed.

Table 12 contains a summarized version of the results of this simulation.

The algorithms, for both scenarios, followed the same trend that each index did in the analyzed period, between 13 September 2021 and 28 September 2021. The AAPL index decreased 3% in value, while the TSLA increased 9.1% as shown by the simulation baseline. The AAPL ROI was higher than the baseline but still ended in negative profit. Meanwhile, the TSLA ROI was smaller than the baseline but ended with positive profit. These results indicate that the model ended up smoothing the risk associated with the investment.

## 6. Discussion of the Results

These results clarify which techniques work better in this specific situation and are a study case for both NLP in Deep Learning as well as for the usage of Deep Learning to process Big Data datasets. However, it is clear that some more study and more fine tuning is needed to better understand the possibilities of using these techniques. 

**Figure 17:**
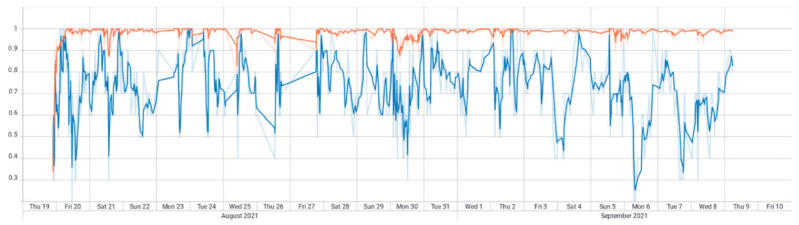
*CNN*-*LSTM* with Stock Indicators accuracy (the orange line represents accuracy on training data and the blue line accuracy on validation data).

Figure 17 shows the *CNN*–*LSTM* SI model training pattern (over 20 days). Accuracy on training data (orange line) stabilized near 100%. Accuracy on validation (blue) data was much more volatile. This may be a symptom of the training strategy used, where new data were added in batches, instead of providing the full dataset from the beginning. The accuracy on test data was 73%—the highest in all models. This graphic shows an irregular behavior on validation data, dipping frequently, which suggests overfitting issues during training. 

**Figure 18:**
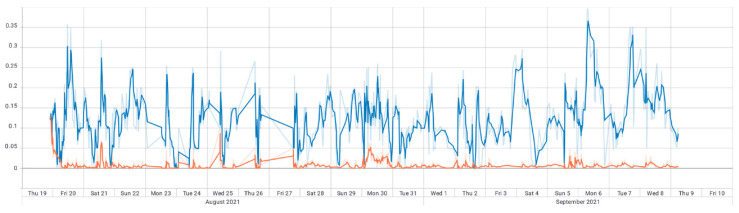
*CNN*-*LSTM* with stock indicators loss (the orange line represents loss on training data and the blue line loss on validation data).

To support this theory, we have the loss function for the same model (where the line colors represent the same datasets as in the accuracy graph) in Figure 18. A non-overfitted model would have both the validation as the training graphs converging towards 0 error, which is not the case. 

Considering all the results presented above, it was possible to conclude that some DL algorithms demand unfeasibly complex data structures, which are too big to cache in RAM. This can be avoided by separating data in batches, using them to train the networks in incremental intervals. This method is the cause of the higher training time associated with the *CNN*-*LSTM SI* algorithm, which is a clear outlier when compared to the rest. This could influence the construction of a neural network, as well as the method used to train it. In this case, this technique was used since the algorithm acts as a time series classifier.

**Figure 19:**
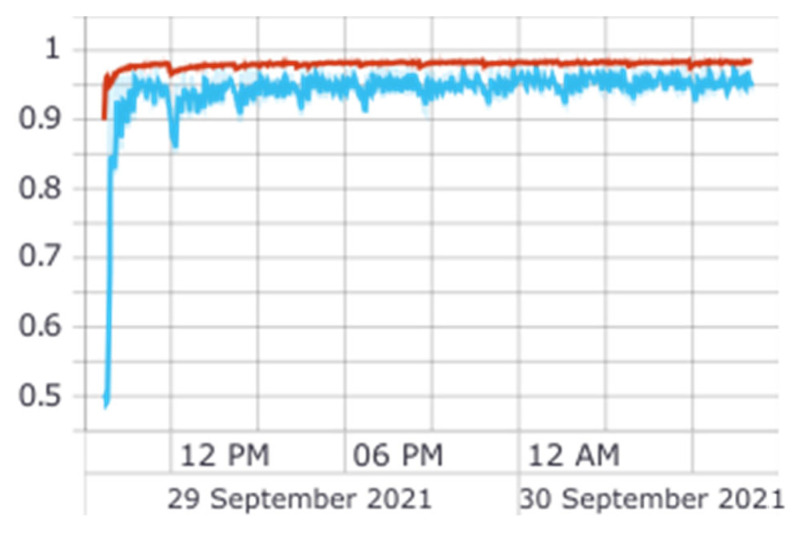
*GRU* train accuracy (the red line represents accuracy on training data and the blue line accuracy on validation data).

Figure 19 shows an example of a different network, which was trained with the traditional Nested Cross Validation method. We can see there are some spikes in the blue line (validation data), which are much smaller than those in the *CNN*-*LSTM SI* graphic, which seems to indicate that this network did not overfit. This may have to do with a better selection of hyperparameters, a less complex input, or even the fact that the model had the whole dataset available (divided into train and validation several times) during the training phase, opposed to the *CNN*-*LSTM SI*, where the data available increased with the epochs. 

When comparing the test data results, the average accuracy results penalize the *CNN*-*LSTM SI*, possibly due to overfitting during training, even though it was the best performer in training and in isolated tests (testing the AAPL and TSLA datasets). It was also the network with the highest standard deviation in terms of result metrics. This could indicate that further hyperparameter tuning or even adjusting the network is necessary, by adding more memory layers (*LSTM*) after Convolutional and Pooling layers, so that older information is not discarded in favor of new information in the batch progression. 

The simulation shows the potential of the models, which were able to both decrease the profits in relation to the baseline and at the same time decrease losses. The simulation was run from 13 September 2021 to 28 September 2021, which is an objectively small timeframe. Running the simulation for a longer period could yield different results and could allow an analysis of the model regarding whether it is correctly predicting sentiment or just guessing. 

Reddit and Twitter data used in the test and simulation phases were not properly moderated by specialists in the subject in analysis. This may have influenced the results obtained by analyzing this data, since there is no proven correlation between the stock values and the sentiments in both these sources. However, the data seemed to have some value, since the simulation, performed using a DL model trained with them, returned a profit in one of the scenarios and smoothed the loss in the other.

## 7. Conclusions and Future Work

In this paper, we have provided an overview on the usage of Deep Learning on unstructured data and how to process Big Data efficiently using specialized hardware techniques. We described how different techniques of Deep Neural Networks act on sentence data producing Sentiment and how numerical data can enrich the techniques used on sentiment data analysis. 

For stock market values, this work contributes by describing different methods of DNNs and their outputs for this type of data. A generalized DL-based classification framework for stock market Sentiment Analysis was proposed. This work also included the study, the development, and the implementation of an automatic classification system based on DL and the validation of its adequacy and efficiency in any scenario, particularly stock market Sentiment Analysis. An assessment of Big Data management, processing, and extraction techniques were also presented. We also explained how to extract, transfer, load, store, and pre-process Big Data. 

In upcoming work, the usage of sentiments by professional stock traders may provide more accurate results and improve classification performance. Using other contexts for training would also provide different results, since these models have been trained in a single stock index context (DJIA), with sentiment obtained from Reddit news headlines data. DJIA is an aggregate of different stock value indices, which makes it a stable stock, with small value changes. This also means that its value usually increases with time, influencing the algorithms as well, creating a bias that all stock indices will increase eventually. The experimental results using a Deep Learning model demonstrated a profit in one of the scenarios and smoothed the loss in the other. Therefore, this work improves stock market prediction using Deep Learning.

As future work, we intend to use data streaming technology to create near real-time models. In addition, we propose the implementation of multiple nodes for DFS as well as Spark.

## Figures and Tables

**Table 1:** Comparison of DNN usage.

	Image Processing	NLP	Dimensionality Reduction
Auto Encoder			✓
Convolutional NNs	✓	✓	✓
Long-Short Term Memory		✓	✓
Gated Recurrent Units		✓	✓
Recursive NNs		✓	✓

**Table 2:** *CNN* hyperparameters.

Hyperparameter	Value
Number of Hidden Layers	8
Dropout	None
Activation function—Conv1D	ReLU
Learning Rate	$1\times 10^{-4}$
Epochs	60
Batch Size	200

**Table 3:** *LSTM* hyperparameters.

Hyperparameter	Value
Number of Hidden Layers	10
Dropout	20%
Activation function—*LSTM*	TanH
Activation Function—*Dense*	ReLU/Sigmoid (last hidden layer)
Learning Rate	$1\times 10^{-2}$
Epochs	150
Batch Size	300

**Table 4:** *GRU* hyperparameters.

Hyperparameter	Value
Number of Hidden Layers	6
Dropout	50%
Activation Function—*GRU*	TanH
Activation Function—*Dense*	ReLU/Sigmoid (last hidden layer)
Learning Rate	$1\times 10^{-3}$
Epochs	150
Batch Size	300

**Table 5:** *CNN*-*GRU* and *CNN*-*BiGRU* Hyperparameters.

Hyperparameter	Value
Number of Hidden Layers	7
Dropout	None
Activation Function—*GRU*	TanH
Activation Function—Conv1D	ReLU
Activation Function—*Dense*	Sigmoid
Learning Rate	$1\times 10^{-3}$
Epochs	150
Batch Size	300

**Table 6:** *LSTM*-*CNN* hyperparameters.

Hyperparameter	Value
Number of Hidden Layers	9
Dropout	None
Activation Function—*LSTM*	TanH
Activation Function—Conv1D	ReLU
Activation Function—*Dense*	ReLU /Sigmoid (last layer)
Learning Rate	$1\times 10^{-2}$
Epochs	150
Batch Size	300

**Table 7:** *CNN*-*LSTM* SI hyperparameters.

Hyperparameter	Value
Number of Hidden Layers	15
Dropout	None
Activation Function—*LSTM*	TanH
Activation Function—Conv1D	ReLU
Activation Function—*Dense*	ReLU/Softmax/Sigmoid
Learning Rate	$1\times 10^{-8}$
Epochs	150
Batch Size	1

**Table 8:** Collected dataset description.

Subset	Number of Sentences	Number of Days	Origin
AAPL	21,936	12	Twitter
BTC	14,600	18	Twitter
DJIA	12,640	12	Twitter
TSLA	22,800	12	Twitter
Stocks	21,132	25	Reddit
Stock Market	21,203	28	Reddit
Wallstreetbets	20,270	15	Reddit
All reddit data	62,605	28	Reddit

**Table 9:** Stock indicator formulas.

Feature	Formula	Feature	Formula
Stochastic %K	$\frac{C_{t}-LL_{n}}{HH_{n}-LL_{n}}$	William’s %R	$\frac{H_{n}-C_{t}}{H_{n}-L_{n}}\times 100$
Stochastic %D	$\frac{\sum_{i=0}^{n-1}\%K_{t-1}}{n}$	A/D Oscillator	$\frac{H_{t}-C_{t-1}}{H_{t}-L_{t}}$
Momentum	$C_{t}-C_{t-n}$	Disparity 5	$\frac{C_{t}}{MA_{5}}\times 100$
Rate of Change	$\frac{C_{t}}{C_{t-n}}\times 100$		

**Table 10:** Training indicators.

Algorithm	Accuracy	Loss	Train Time
*CNN*	0.520	0.125	47 h
*LSTM*	0.644	0.073	14 h
*GRU*	0.534	0.025	22 h
*CNN*-*GRU*	0.632	0.105	16 h
*CNN*-*BiGRU*	0.591	0.093	17 h
*CNN*-*LSTM*	0.534	0.233	22 h
*CNN*-*LSTM SI*	0.726	0.125	20 days

**Table 11:** Test indicators.

Algorithm	Accuracy	Specificity	F1 Score	MCC	Precision	Sensitivity	Fallout
*CNN*	0.575	0.011	0.727	−0.001	0.577	0.99	0.989
*LSTM*	0.507	0.396	0.579	−0.003	0.573	0.6	0.604
*GRU*	0.506	0.449	0.551	0.02	0.574	0.57	0.551
*CNN*-*GRU*	0.521	0.302	0.625	0.007	0.579	0.704	0.698
*CNN*-*BiGRU*	0.518	0.331	0.611	−0.004	0.574	0.665	0.669
*CNN*-*LSTM*	0.525	0.409	0.597	−0.022	0.596	0.601	0.591
*CNN*-*LSTM SI*	0.486	0.676	0.366	0.03	0.489	0.316	0.324

**Table 12:** Summary of the simulation.

Simulation	Baseline	ROI	Model Strategy	Model Strategy ROI
TSA	1091.49$	9.1%	1043.95$	4.4%
AAPL	970.09$	−3.0%	979.04$	−2.1%
